# Improved Correction of Atmospheric Pressure Data Obtained by Smartphones through Machine Learning

**DOI:** 10.1155/2016/9467878

**Published:** 2016-07-25

**Authors:** Yong-Hyuk Kim, Ji-Hun Ha, Yourim Yoon, Na-Young Kim, Hyo-Hyuc Im, Sangjin Sim, Reno K. Y. Choi

**Affiliations:** ^1^Department of Computer Science and Engineering, Kwangwoon University, 20 Kwangwoon-ro, Nowon-gu, Seoul 01890, Republic of Korea; ^2^Department of Embedded Software Engineering, Kwangwoon University, 20 Kwangwoon-ro, Nowon-gu, Seoul 01890, Republic of Korea; ^3^Department of Computer Engineering, College of Information Technology, Gachon University, 1342 Seongnam-daero, Sujeong-gu, Seongnam-si, Gyeonggi-do 13120, Republic of Korea; ^4^Korea Oceanic and Atmospheric System Technology, No. 1503, STX W-Tower, 90, Gyeongin-ro 53-gil, Guro-gu, Seoul 08215, Republic of Korea; ^5^Observation Research Division, National Institute of Meteorological Sciences, 33 Seohobuk-ro, Seogwipo-gi, Jeju-do 63568, Republic of Korea; ^6^Geography and Environment, University of Southampton, University Road, Southampton SO17 1BJ, UK

## Abstract

A correction method using machine learning aims to improve the conventional linear regression (LR) based method for correction of atmospheric pressure data obtained by smartphones. The method proposed in this study conducts clustering and regression analysis with time domain classification. Data obtained in Gyeonggi-do, one of the most populous provinces in South Korea surrounding Seoul with the size of 10,000 km^2^, from July 2014 through December 2014, using smartphones were classified with respect to time of day (daytime or nighttime) as well as day of the week (weekday or weekend) and the user's mobility, prior to the expectation-maximization (EM) clustering. Subsequently, the results were analyzed for comparison by applying machine learning methods such as multilayer perceptron (MLP) and support vector regression (SVR). The results showed a mean absolute error (MAE) 26% lower on average when regression analysis was performed through EM clustering compared to that obtained without EM clustering. For machine learning methods, the MAE for SVR was around 31% lower for LR and about 19% lower for MLP. It is concluded that pressure data from smartphones are as good as the ones from national automatic weather station (AWS) network.

## 1. Introduction

Severe weather, such as local torrential rains, gusts, or environmental disasters, is being found more frequently in recent years. Public warnings and alerts on the basis of near real-time observation are therefore increasingly important especially for highly populated cities. Large numbers of observations are to be made in the area of interest for monitoring weather-related events, but their spatial resolution from conventional national scale network of automatic weather stations (AWSs) is often insufficient. Although several studies [1–4] have been conducted for feasibility of portable meteorological equipment to enhance weather observation and forecast, increased use of portable meteorological equipment is still limited due to geographic constraints and financial reasons.

The advent of microelectromechanical systems (MEMS) sensors opened up new possibilities in the field of weather observation. Smartphones have been widely equipped with these devices, whose performance has also been improving quickly in response to user demand. Potential candidate MEMS-based sensors in most smartphones for meteorological observations are atmospheric pressure, temperature, and relative humidity. Thus, it is expected that smartphones may be used to obtain more specific meteorological data at a low cost, even if only for some basic weather variables. However, other studies [5, 6] have pointed out that issues of sensor performance and data reliability need to be resolved in order to utilize data obtained by sensors in smartphones.

In a previous study, we proposed a correction method that minimizes errors between the data obtained by smartphones and meteorological data of the Korea Meteorological Administration (KMA) by collecting the data from MEMS meteorological sensors built into smartphones using an application called* Yeowoobi* [7], which can obtain such data from smartphones with Android OS 4.0 or greater and store them in a separate server. There have been several studies or guidelines published [8–10] on error correction of public meteorological equipment, a study [11] on the analysis of air temperature by using battery temperature measurements in smartphones, and a study [12] on observation of surface pressure, but our previous study was the first to use smartphones to correct atmospheric pressure data.

Our current study is intended to enhance the correction method used in our previous study by classifying the data previously obtained according to time, considering human mobility patterns, and using various machine learning methods. Data obtained and preprocessed in the same manner as in our previous study [7] were classified according to time (daytime or nighttime and weekday or weekend) based on user behavior patterns. They were automatically reclassified through clustering, and various machine learning methods such as linear regression (LR), multilayer perceptron (MLP), and support vector regression (SVR) were applied to them in order to analyze the results for comparison. Each machine learning method was established by identifying a parameter value leading to the optimal result, and the time required for determining this parameter value was also considered.

This paper is organized as follows.  Section 2 introduces the machine learning methods used to improve the existing correction method in this study; Section 3 describes the meteorological data used in this study as well as the quality control (QC) preprocessing and the classification of data by time to compare the results with those from the previous study; Section 4 identifies the method that exhibits the best performance by analyzing the results of using various machine learning methods (i.e., clustering, LR, MLP, and SVR) based on data in the fields added; Section 5 analyzes the experimental results; and Section 6 presents considerations and directions for future work.

## 2. Machine Learning

WEKA [13] is a machine learning program developed by the University of Waikato in New Zealand enabling the user to analyze data and to perform prediction modeling by using various machine learning algorithms. In this study, WEKA was used for data analysis by applying LR, MLP, SVR, and expectation-maximization (EM) clustering algorithms. These algorithms are described briefly in the subsections that follow.

### 2.1. Linear Regression

LR is a regression analysis method used for modeling a linear relationship between more than one independent variable and a dependent variable. It combines weights whose initial values are provided and data attributes to represent each layer in the form of a linear equation. The predicted value ${\hat{x}}^{\left(i\right)}$ of the *i*th layer can be represented as

(1) $$\begin{array}{c} {\hat{x}}^{\left(i\right)}=\omega_{0}a_{0}^{\left(i\right)}+\omega_{1}a_{1}^{\left(i\right)}+\omega_{2}a_{2}^{\left(i\right)}+\cdots +\omega_{k}a_{k}^{\left(i\right)}=\overset{k}{\underset{j=0}{\sum }}\omega_{j}a_{j}^{\left(i\right)}. \end{array}$$

Weights (*ω*_*j*_s) are derived from the number *n* of learning data. The difference between the calculated predicted value and the actual value is calculated by (2) as well as weights (*ω*_*j*_s) that minimize the difference to derive an LR equation:

(2) $$\begin{array}{c} \overset{n}{\underset{i=1}{\sum }}{\left(\;x^{\left(i\right)}-{\hat{x}}^{\left(i\right)}\;\right)}^{2}. \end{array}$$

### 2.2. Multilayer Perceptron

A multilayer neural network [14] is a nonlinear classification method based on Perceptron, which is a linear classifier, but unlike the existing Perceptron, it has a hidden layer between the input layer and the output layer. Learning in a multilayer neural network can be roughly divided into two stages. The first stage is a forward computation that calculates a predicted value from the input layer to the output layer, and the second stage is an error backpropagation that renews weights to minimize the error between the predicted value and the actual value. Given a multilayer neural network that has *p* node(s) in one hidden layer, *n* nodes in the input layer, and *m* nodes in the output layer, a forward computation is performed using (3) to calculate from the input layer to the hidden layer and (4) to calculate from the hidden layer to the output layer:

(3) zj=τ∑i=1nxiuij+u0jj=1,2,…,p,

(4) ok=τ∑j=1pzjvjk+v0kk=1,2,…,m,

where *τ*(·) is an activation function. Typically, a sigmoid function as shown in (5) is the most widely used, and a gradient is determined according to the *α* values:

(5) $$\begin{array}{c} \tau \left(\;x\;\right)=\frac{1}{1+e^{-\alpha x}}. \end{array}$$

The error (*E*) between the value *o* obtained through the forward computation and the actual value *t* is defined as shown in

(6) $$\begin{array}{c} E=\frac{1}{2}\overset{m}{\underset{k=1}{\sum }}{\left(\;t_{k}-o_{k}\;\right)}^{2}. \end{array}$$

The error backpropagation process, which renews weights *v* and *u* in order to reduce *E*, is repeated for each generation *h* through

(7) vh+1=vh+Δv,uh+1=uh+Δu.

The MLP equation is derived by using the optimal weights obtained through the process above.

### 2.3. Support Vector Regression

SVR is a support vector machine (SVM) algorithm that is used to solve regression problems and that can also be applied in nonlinear prediction. In contrast to the existing algorithms, including neural networks, it leads to the optimized generalization performance by maximizing a space that exists between two layers.

Instances that are the most adjacent to a hyperplane that has the maximum space or instances located the shortest distance from a plane are called support vectors. Only one set of these support vectors determines the hyperplane that has the maximum space regarding a learning problem; the other instances are irrelevant to learning. The SVR equation is shown as

(8) $$\begin{array}{c} f\left(\;x\;\right)=\overset{l}{\underset{i=1}{\sum }}\alpha_{i}t_{i}K\left(\;x_{i}\cdot x\;\right)+b. \end{array}$$

All the results calculated by using a kernel function *K* and a test sample *x* for every *x*_*i*_ having *l* support vector(s) are added together. *α* is a Lagrange multiplier, *t* is an integer that represents the category, and *b* is a constant that represents the location on the hyperplane.

In addition, the SMOreg algorithm is a kind of SVR based on the sequential minimal optimization (SMO) algorithm, which is an optimization algorithm proposed to use SVR [15]. Whereas an inefficiency problem is caused in the SMO algorithm because there is only one threshold, this problem is solved in the SMOreg algorithm by using two thresholds [16].

### 2.4. Expectation-Maximization Clustering

EM clustering is an iterative algorithm that first estimates initial values for unobservable parameters and then calculates the cluster probability of each instance by using the initial values to find the parameter value having the maximum likelihood [17].

First, after initial values for the parameters in each cluster have been assigned, the probability *P*(cluster∣instance) for each instance to be included in clusters is calculated. Then, parameters that have the maximum likelihood are recalculated by using the instance points included in each cluster. This process is performed repeatedly until the parameter values for each cluster do not change.

## 3. Experimental Data

### 3.1. Smartphone Data

Meteorological data for South Korea for dates between July 1 and December 31 in 2014 were obtained using a smartphone application called* Yeowoobi* (the term* Yeowoobi* means sunshower in Korean). The meteorological data collected by this application include the time at which data are received at a server, transmission methods, location precision time information (i.e., year, month, day, hour, minute, and second), latitude (degrees), longitude (degrees), spot atmospheric pressure (hPa), user identification number, temperature (°C), relative humidity (%), and smartphone information. The initial cycle for obtaining data for atmospheric pressure, temperature, and relative humidity takes 10 minutes. Users of the* Yeowoobi* app select one of nine stages for an observation cycle (from one minute to three hours) based on various factors such as battery consumption and cost of the data transfer.

**Figure 1:**
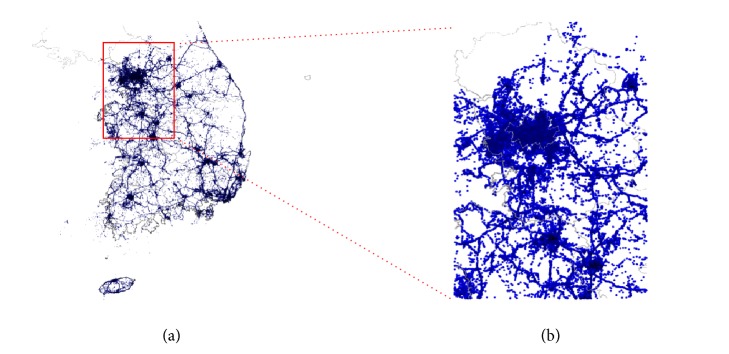
Distribution of observation data obtained by smartphones. South Korea (a) and Gyeonggi-do subset (b).

In this study, the subset of meteorological data obtained in Gyeonggi-do (including Seoul) was used as the experimental data. When latitude and longitude are calculated to the third decimal point, the number of data obtained is approximately two million (47% of the entire data set collected), and the number of users is approximately two thousand (63% of the entire set of users) (Table 1).  Figure 1 shows a map of the observation data across South Korea obtained by smartphones, showing location information for the observation data in Gyeonggi-do. In addition, among 692 points of public meteorological equipment throughout the country, 238 points are located in Gyeonggi-do; 53 of these 238 sites correspond to locations where data were also collected by smartphones.

**Figure 2:**
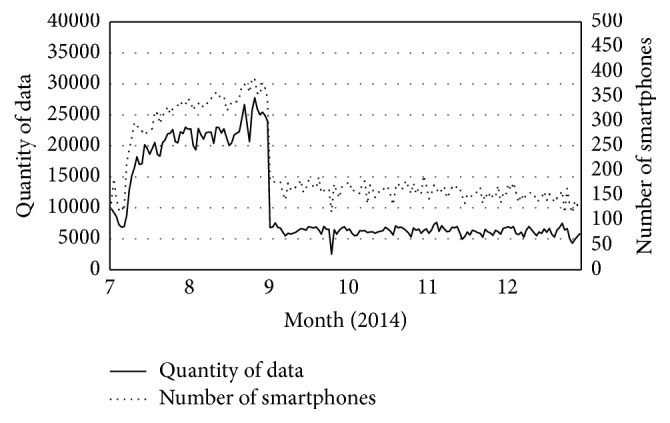
The number of smartphone users and quantity of meteorological data for Gyeonggi-do.

Figure 2 shows changes in the number of smartphone users and in the quantity of meteorological data obtained by smartphones in Gyeonggi-do across time. As the number of smartphone users increased to nearly 400 per day from July to the middle of August, the quantity of observation data obtained also increased, to nearly 30,000 per day. Then, after September, the number of smartphone users decreased to around 150 to 200 per day and the quantity of observation data to approximately 5,000 per day; this sudden decrease occurred because the* Yeowoobi* app was not advertised any more. During the advertisement of the* Yeowoobi* app, we had done an event of giving each new user a small gift in compensation for using the app. It is regarded that the number of users constantly increased during the event period but inconveniently it rapidly decreased concurrently with the stop of the ad event.

As detailed in our previous study [7], the preprocessing of the data was performed in three stages, including a physical limit test to remove values beyond a physical threshold according to the standard regulation for weather observation by the World Meteorological Organization (WMO) [18], reduction to mean sea level using digital elevation model (DEM) data [19], and removal of abnormal values (i.e., those beyond 3*σ*).

### 3.2. Data Obtained by Public Meteorological Equipment

Data obtained by automatic weather stations (AWSs) and automated synoptic observing systems (ASOSs), which are operated by the Korea Meteorological Administration, were used as public meteorological data. As of May 2015, the number of these installations of observation equipment across South Korea was 692, with 238 of these being located in Gyeonggi-do. Of the entire set of observation equipment installations in Korea, 256 can obtain atmospheric pressure data. That is because whereas five elements (i.e., wind direction, wind speed, temperature, precipitation, and rainfall occurrence) were the main observation items until 2007, the element of atmospheric pressure was added to 100 installations that were replaced after 2007, and the element of relative humidity was added to equipment replaced from 2010 onward. The collection period of observation data for the AWS is one minute, and the observation resolutions of the ASOS and the AWS are approximately 36.0 km and 13.3 km, respectively [20].

### 3.3. Classification according to Time

**Figure 3:**
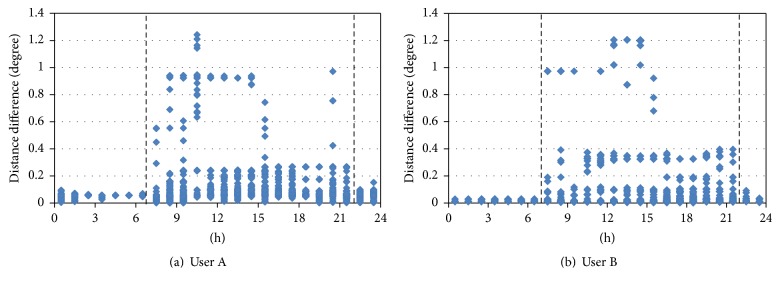
Distribution of distance differences (degrees) of Users A and B from their respective average positions according to time of day.

Figure 3 shows the distribution of distance differences of Users A and B, two representative users, from their respective average positions according to time of day by using location data for each user in order to examine the mobility of smartphone users (personal information of the users is not obtained). The sample sizes of the collected data from Users A and B are 24,430 and 19,272, respectively. For both users, the distance difference did not exceed 0.2 degrees between 10 p.m. and 7 a.m., but it became greater than 0.2 degrees between 7 a.m. and 10 p.m. In other words, the scope of movement for both users was either narrow or unlikely to exist between 10 p.m. and 7 a.m., and it became significantly wider between 7 a.m. and 10 p.m.

**Figure 4:**
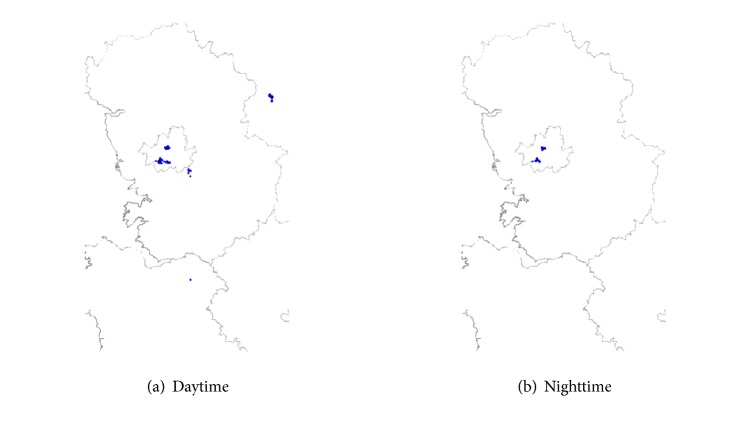
Location distribution of User C in Gyeonggi-do in October and November of 2014.

Based on this result, classification by time of day was performed by defining the time range between 7 a.m. and 10 p.m. (during which smartphone users were active) as the daytime and that between 10 p.m. and 7 a.m. of the following day (during which the users were expected to be less active) as the nighttime.  Figure 4 shows the location distribution for a random user (User C), who performs activities mainly in Gyeonggi-do, during the daytime and during the nighttime, demonstrating that he or she shows more changes of location during the daytime than during the nighttime.

Table 2 shows the comparison of the weighted mean value of the results obtained by performing linear regression analysis based on classification by data source (AWS or smartphone) in Gyeonggi-do and the weighted mean values of the results obtained by performing linear regression analysis where classification by time of day (daytime or nighttime) is added. For each linear regression analysis result, 212 users had 1,000 observations or more, and the data from these users were used for the calculation of the mean values. Compared to the mean absolute error (MAE) derived by applying classification by data source, the MAE derived by applying classification to the daytime data was slightly higher, and that for the nighttime data was markedly lower. In summary, for both the MAE and the root-mean-squared error (RMSE), the daytime figure was approximately twice the nighttime figure.

**Figure 5:**
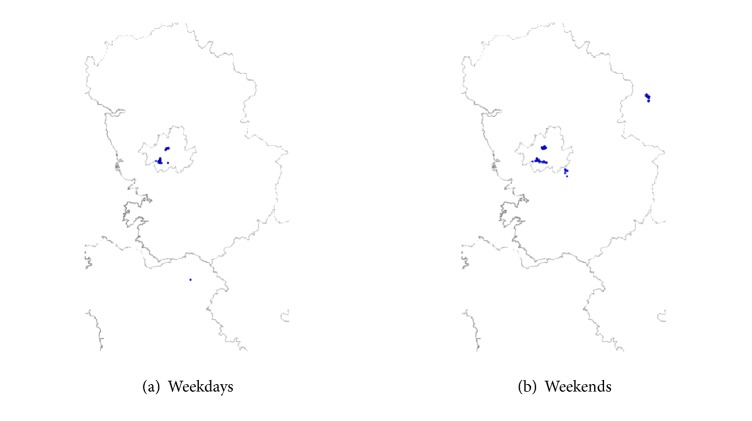
Location distribution of User C in Gyeonggi-do in October and November of 2014.

In addition to classifying observations according to time of day (daytime or nighttime), we further classified them according to day of the week (weekday or weekend).  Figure 5 shows the location distribution of User C on weekdays and weekends. User C shows a consistent movement pattern that is kept within certain bounds on weekdays but on weekends shows an irregular movement pattern with a distribution of points greater than on weekdays.

Table 3 shows the weighted mean values obtained from linear regression analysis based on classification into daytime-weekdays, daytime-weekends, nighttime-weekdays, and nighttime-weekends by adding the classification into weekdays and weekends to the existing classification into daytime and nighttime. Only users having 1,000 or more observations were used to obtain the mean values in the case of the daytime data, and only users having 500 or more observations were used to obtain the mean values in the case of the nighttime data.

The results of performing linear regression analysis based on classification into daytime and nighttime and into weekdays and weekends according to data source (AWS or smartphone) indicate that the MAE and RMSE for daytime-weekends were lower than those for the daytime-weekdays. In addition, within the nighttime data, the MAE was lower for weekends than for weekdays, similar to that for the daytime data, whereas the RMSE was higher for weekends.

When the nighttime data were classified into nighttime-weekdays and nighttime-weekends, the quantity obtained became scarce, and there was no significant difference found between the two categories of nighttime data. Thus, nighttime data obtained without further classification into weekdays and weekends are used in the following experiments.

## 4. Data Analysis Using Machine Learning

Data were analyzed by using machine learning methods included in WEKA, and the MAE and RMSE values were compared. Tenfold cross validation was used as a method of training and as a test for model verification. Data obtained by smartphones and at public meteorological equipment installations were used as the learning data (refer to the Appendix for details of data fields), and values of mean sea level pressure (MSLP) obtained as public meteorological data at points that were closest spatially and temporally were used as true values.

### 4.1. Regression Analysis

A user near number 649 point who has 16,897 observations, which include the largest quantity of daytime-weekday data, was selected, and the 10,000 latest observations from this user were extracted to be used for testing. The data were selected in groups in multiples of 1,000, and MAE values were obtained and compared by applying various regression analysis methods supported by WEKA to them.

**Figure 6:**
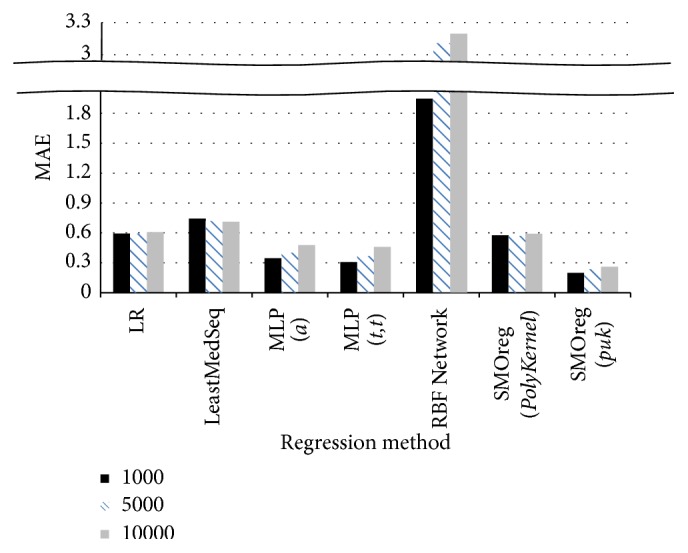
Comparison of results according to regression analysis method.

Figure 6 shows the comparison of the results of regression analysis when the quantity of data was 1,000, 5,000, and 10,000, respectively. The MAEs for RBF Network [21] were approximately 2 to 3, and the MAEs of Least Median Squared Linear Regression (LeastMedSeq) [22] were approximately 0.7, both sets of which were relatively high compared to the MAEs for LR, MLP, and SMOreg, each of which was 0.6 or less. For the MLP method, similar results were obtained for the MAEs when there was one hidden layer and the number of nodes in the hidden layer was (number of attributes + number of layers)/2 (Option *a*) and for the MAEs when there were two hidden layers and the number of nodes in the hidden layers was the number of attributes + the number of layers (Option *t*, *t*). For the SMOreg method, the MAEs obtained using Pearson Universal Kernel (Option* puk*) showed twice better performance than those obtained using Polynomial Kernel (Option* PolyKernel*).

**Figure 7:**
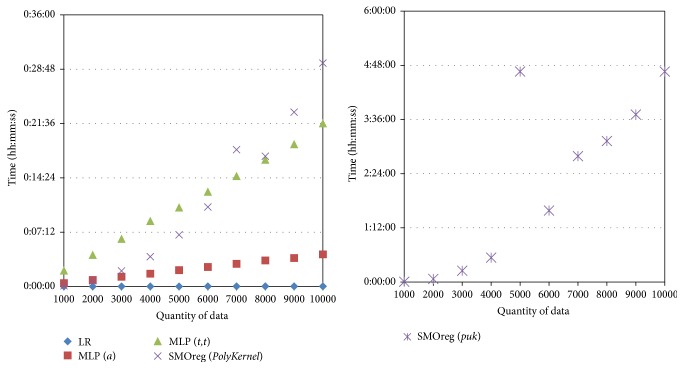
Computing times for LR, MLP, and SMOreg according to the quantity of data.

Figure 7 shows the comparison of the computing times for LR, MLP, and SMOreg. Although the MAEs for LR were higher than those of the others, LR's computing times were very fast: one second on average. In contrast, SMOreg (Option* puk*), which had the lowest MAEs, had relatively long computing times. For MLP, the MAEs using Options *a* and *t*, *t* were similar, whereas the execution times using Option *a* were shorter than those using Option *t*, *t*. Based on these results, Option *a* is used for MLP and Option* puk* for SMOreg in the following experiments.

### 4.2. Clustering

**Figure 8:**
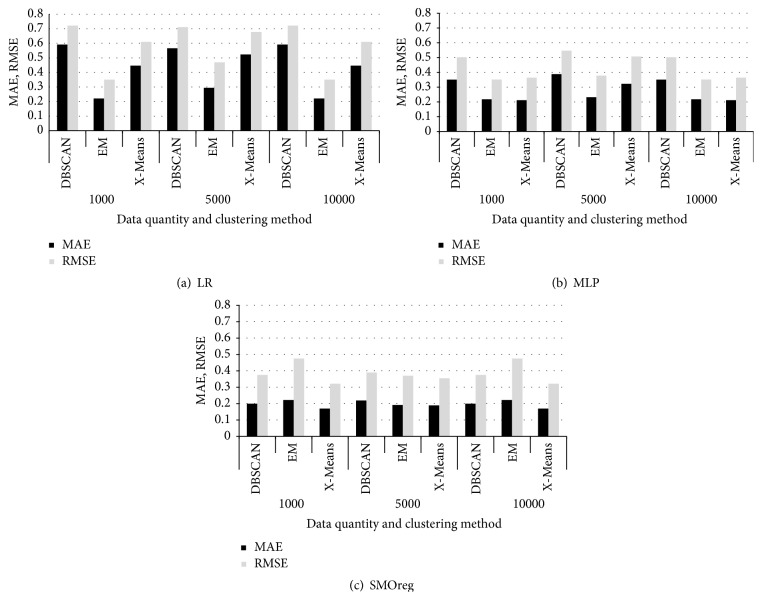
Comparison of weighted mean MAEs and RMSEs obtained by applying LR, MLP, and SMOreg through DBSCAN [23], EM, and X-Means [24] clustering.

From the 10,000 observations, the latest data were selected and were clustered in groups of multiples of 1,000 using clustering methods in WEKA. Then, LR, MLP, and SMOreg were performed on them in order to compare the MAEs and RMSEs.  Figure 8 shows the results obtained by each method when the quantities of data were 1,000, 5,000, and 10,000, respectively. The data given in Section 4.1 were also used, and only a subset of fields were used (latitude; longitude; altitude; DEM altitude; atmospheric pressure measured by smartphones; atmospheric pressure corrected; distance between smartphone and nearest AWS; and mean sea level pressure, temperature, and relative humidity measured by AWSs) from among the entire set of fields available in order to reduce the clustering computing time. All the clustering processes were performed using default parameter values established in WEKA.

When LR was applied, the MAEs for EM were lower than those for DBSCAN and X-Means. When MLP was applied, the MAE for EM (being approx. 0.2) was also lower than those for the other two methods when the quantity of data was 5,000; when the quantity of data was 1,000 or 10,000, values for EM were lower than those for DBSCAN and were similar to those for X-Means. As for SMOreg, the MAEs for X-Means were the lowest whether the quantity of data was 1,000, 5,000, or 10,000, but the overall MAE value was approximately 0.2 and showed an insignificant difference between clustering methods.

**Figure 9:**
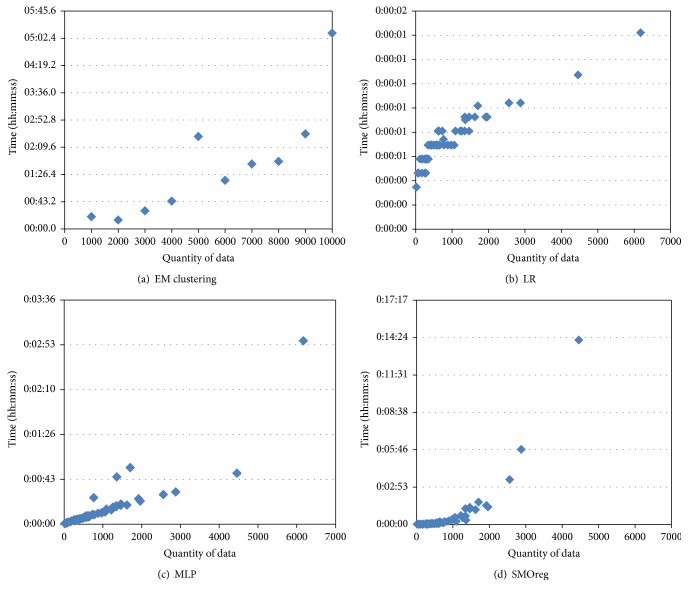
Computing time for EM clustering according to the quantity of data (a) and computing times for LR (b), MLP (c), and SMOreg (d) after EM clustering according to the quantity of data.

**Figure 10:**
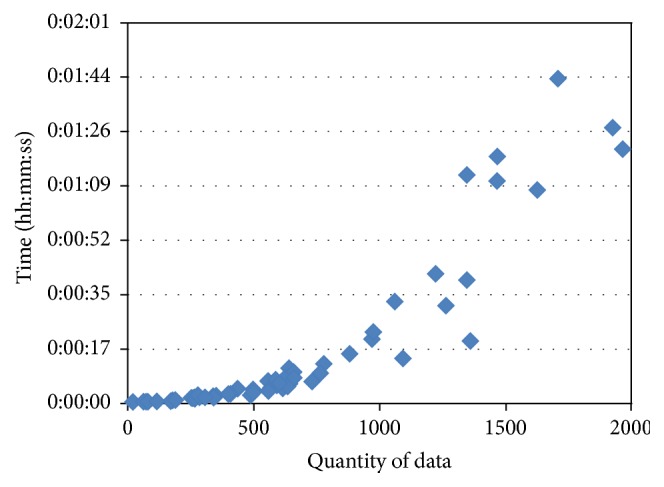
Computing time of SMOreg according to the quantity of data (partial magnification of Figure 9(d)).

Figure 9 shows the computing times for machine learning according to the quantity of data. The computing time for EM clustering was one minute or less when the quantity of data was 4,000 or less, but it increased linearly to between two and three minutes when the quantity of data was 5,000 or greater and increased rapidly to five minutes when the quantity of data was 10,000. Computing time for LR after EM clustering increased as the quantity of data increased, but it showed very high speed overall, under two seconds. Computing time for MLP was one minute or less when the quantity of data was 5,000 or less, but it increased rapidly to three minutes when the quantity of data was approximately 6,000. Computing time for SMOreg increased exponentially as the quantity of data increased (Figure 10); it required 15 minutes for computation when the quantity of data was approximately 4,500.

**Figure 11:**
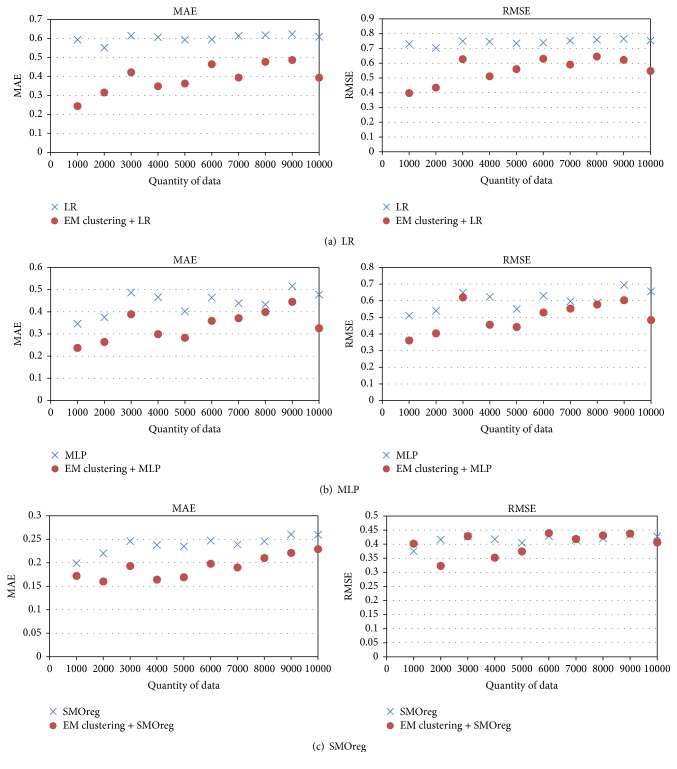
Comparison between the regression analysis results when EM clustering was performed and those when EM clustering was not performed.

Figure 11 shows the comparison of the results of regression analysis derived through EM clustering and those derived without EM clustering. The MAEs obtained from regression analysis through EM clustering was lower by 73% on average than those of regression analysis without EM clustering.

## 5. Results

We analyzed the results presented in Sections 3 and 4, which were based on the data obtained by smartphones between July and December 2014 in Gyeonggi-do. After classifying the entire set of data into daytime-weekday, daytime-weekend, and nighttime groups, the data from users having 1,000 or more observations were extracted. Then, only the last 1,500 observations were used for regression analysis when the quantity of data was between 1,000 and 5,000. When the quantity of data was 5,000 or greater, EM clustering was used. If the quantity of data was 1,500 or greater in each cluster, the latest 1,500 data were selected; if the quantity of data was less than 1,500, regression analysis was performed on all the corresponding data.

Table 4 shows the experimental environment and summary statistics. EM clustering, LR, MLP, and SMOreg were conducted using the WEKA program. Tenfold cross validation was used in training and testing, which are for model verification. The entire computing time in the single CPU was 20:54:11 (hh:mm:ss), most of which was used for training. When it is assumed that a model is generated once a month, it is sufficient to perform real-time correction. Data from 279 users were analyzed, and the locations where data were collected corresponded to the 26 AWS points. The total number of models was 670, and the average number of data samples for each model was 1,234.

Table 5 shows the results of regression analysis through EM clustering, with MAEs and RMSEs calculated as the weighted mean values. MAE values are generally 0.5 or lower, and of these, the MAE derived through regression analysis using SMOreg was 0.297 (RMSE 0.556). This value is lower than those for LR and MLP, thus demonstrating that the SMOreg model has the best performance in terms of prediction accuracy.

## 6. Conclusions

Our earlier study [7] for correction of atmospheric pressure data from smartphones has been extended by adding classification according to time (daytime/nighttime and weekday/weekend), that is, considering human mobility patterns, and applying machine learning. Results showed an error tends to be lower in the nighttime than daytime and improved its quality during weekends, which is mainly due to lesser mobility of smartphone users.

Moreover, regression analysis using EM clustering caused the decrease of the MAE by an average of 26% with comparison to that obtained by conducting regression analysis without EM clustering. Regarding the clustering types, EM clustering showed the best performance in terms of prediction accuracy; in regression analyses using EM clustering, the best performance was achieved with SMOreg, followed by MLP, and then LR. The MAE for SMOreg was lower by around 31% than that for LR and by around 19% than that for MLP. As the correction accuracy of atmospheric pressure data has been improved by using the correction method proposed in this study, the pressure data from smartphones can be used as additional information from public meteorological equipment.

Further studies are currently conducted to (i) address problems found in this study by examining a method of reducing computing time for clustering and regression analysis, (ii) develop subminiature meteorological equipment (mini-AWSs) incorporating the correction method proposed in this study and investigate whether they can be used to supplement existing AWSs, (iii) compare errors according to velocity by using smartphone movement based on location data, and (iv) validate the utilization of the proposed method by comparing the results of applying various additional preprocessing steps such as temporal consistency, persistence and step tests, and spatial quality consistency. Additionally, (v) as the collected data are accumulated for quite a long period, we will be able to apply the presented correcting method by classifying the data according to other criteria such as seasons of the year and weather elements (e.g., precipitation), and these trials will be meaningful.

## Figures and Tables

**Table 1:** Meteorological data obtained by smartphones.

Category	South Korea	Gyeonggi-do
Time range	From July 1, 2014, to December 31, 2014 (184 days)	
Location range		
Latitude	33.203–38.575°N	36.394–38.283°N
Longitude	125.287–129.576°E	126.379–127.858°E
Number of users	3,053	1,927
Number of observations	4,257,430	1,998,935

**Table 2:** Comparison of weighted mean values between the case using Gyeonggi-do data from users having 1,000 or more observations and applying a linear regression analysis by data source (AWS or smartphone) and the case using Gyeonggi-do data from users having 1,000 or more observations and applying a linear regression analysis with the additional classification into daytime and nighttime.

Category	Weighted mean MAE	Weighted mean RMSE
Classified by data source (AWS or smartphone)	0.508	1.051
Classified by data source		
Daytime	0.589	0.905
Nighttime	0.286	0.583

MAE: mean absolute error; RMSE: root-mean-squared error.

**Table 3:** Comparison of weighted mean values between the case applying classification of daytime data from users having 1,000 or more observations and that applying classification of nighttime data from users having 500 or more observations, applying a linear regression analysis based on classification by data source (AWS or smartphone), time of day (daytime or nighttime), and day of week (weekday or weekend) for data in Gyeonggi-do.

Category	Weighted mean MAE	Weighted mean RMSE
Daytime		
Weekdays	0.575	0.878
Weekends	0.512	0.802

Nighttime		
Weekdays	0.283	0.533
Weekends	0.259	0.597

**Table 4:** Experimental environment and summary statistics.

Computer specifications	CPU	Intel Xeon CPU E5-2620 @ 2.10 GHz	
	Memory	8 GB	

Program	WEKA	Version 3.6.10	

Running time (hh:mm:ss)	Clustering	5:24:35	
	LR	0:09:13	
	MLP	5:54:04	
	SMOreg	8:28:54	

Data	Total number of AWS points	26	
	Total number of smartphone users	279	
	Total number of regression models	670	
	Number of samples	Mean	1,234.333/model
		Standard deviation	387.935
	Number of models	Mean	2.401/user
		Standard deviation	2.403
	Number of users	Mean	12.077/point
		Standard deviation	13.683

**Table 5:** Results of regression analysis through EM clustering.

	MAE	RMSE
LR	0.431	0.678
MLP	0.361	0.570
SMOreg	**0.297**	**0.556**

**Table 6:** Data fields used for machine learning.

Data type or source	Field	Unit or format

Observation data obtained by smartphones	Atmospheric pressure, measured	hPa
	Atmospheric pressure, corrected (MSLP)	hPa
	Year	YYYY
	Month	MM
	Day	DD
	Hour	hh
	Minute	mm
	Second	ss
	Latitude	Degrees
	Longitude	Degrees
	Altitude	m
	Precision of location measurement	—
	DEM altitude	m

Observation data obtained by the nearest AWS	AWS number	—
	AWS latitude	Degrees
	AWS longitude	Degrees
	AWS altitude	m
	Mean sea level pressure	hPa
	Temperature	K
	Relative humidity	%

Distance between the smartphone and its nearest AWS	Difference between locations	Degrees

## Appendix

See Table 6.
